# Towards Functional Droplet Architectures: a Belousov-Zhabotinsky Medium for Networks

**DOI:** 10.1038/s41598-018-30819-6

**Published:** 2018-08-23

**Authors:** Kai Ming Chang, Maurits R. R. de Planque, Klaus-Peter Zauner

**Affiliations:** Electronics and Computer Science, University of Southampton, Southampton, SO17 1BJ United Kingdom

## Abstract

The confluence of droplet-compartmentalised chemical systems and architectures composed of interacting droplets points towards a novel technology mimicking core features of the cellular architecture that dominates biology. A key challenge to achieve such a droplet technology is long-term stability in conjunction with interdroplet communication. Here, we probed the parameter space of the Belousov-Zhabotinsky (BZ) medium, an extensively studied model for non-equilibrium chemical reactions, pipetted as 2.5 mm droplets in hexadecane oil. The presence of asolectin lipids enabled the formation of arrays of contacted BZ droplets, of which the wave patterns were characterised over time. We utilised laser-cut acrylic templates with over 40 linear oil-filled slots in which arrays are formed by pipetting droplets of the desired BZ composition, enabling parallel experiments and automated image analysis. Using variations of conventional malonic acid BZ medium, wave propagation over droplet-droplet interfaces was not observed. However, a BZ medium containing both malonic acid and 1,4-cyclohexanedione was found to enable inter-droplet wave propagation. We anticipate that the chemical excitation properties of this mixed-substrate BZ medium, in combination with the droplet stability of the networks demonstrated here for nearly 400 droplets in a template-defined topology, will facilitate the development of scalable functional droplet networks.

## Introduction

A drop of water surrounded by oil can act as a small reaction vessel able to hold hydrophilic compounds. If amphiphilic molecules are present in the oil or in the water phase, they will self-assemble at the water-oil interface resulting in a surfactant-coated aqueous droplet. Such surfactant-stabilised droplets typically do not merge when brought into contact. In case the surfactant is a lipid molecule, a lipid bilayer can be formed at the droplet-droplet contact point. The bio-compatibility of lipid-coated droplets, in combination with the ease of microfluidic mass-production, underpins a flourishing research area. This has resulted in the first practical steps towards functional single droplets and a vision for realising droplet-confined complex chemical machines^1^. In parallel to the advancement in droplet functionality, techniques for the assembly of large-scale structures from droplets are under development with a view towards purposefully designed synthetic tissues comprising hundreds of thousands of specialised functional droplets^2^. While the future of droplet architectures will arguably be dominated by macromolecular biochemistry, it is at present fruitful to use simpler systems in the development phase. A particularly suitable model system is the Belousov-Zhabotinsky (BZ) medium because it is rich enough in functionality, with applications ranging from sensing to actuation^3^, and it is very well studied in bulk and in spatially structured form^4^. Moreover, the light sensing capability of ruthenium catalysed BZ medium and the possibility to halt its oscillation by applying an electric potential open up the possibility to interface with BZ droplet architectures as well as to control individual BZ droplets.

Some microscale structuring methods simply impose physical boundaries on a continuous BZ medium, for example 1,4-cyclohexanedione (CHD) BZ confined in polydimethylsiloxane (PDMS) networks, where the local surface-to-volume ratio affects the extent of bromine diffusion into the PDMS matrix^5^. For water-in-oil microemulsions, BZ medium constitutes the attoliter volume of reverse surfactant micelles, with apolar reaction species diffusing through the oil to adjacent micelles. Typically these emulsions act as a continuous medium, with the oscillation wavelength larger than the compartment size, and give rise to a variety of global patterns^6^. With BZ solution on a surface of light-deactivated catalyst, the medium is again continuous but oscillations only propagate within the active catalyst areas defined by patterned illumination, and thus allows for the control of the contact geometry of the active compartments. Here the compartments are larger than the wavelength and patterns appear within the compartments^7^. Large compartments with physical boundaries can be achieved with surfactant-stabilised droplets-in-oil.

Previous work on networks of BZ droplets-in-oil realised with microfluidic approaches was recently reviewed by Torbensen *et al*.^8^. Epstein and co-workers formed a linear array of surfactant-coated malonic acid (MA) BZ droplets, separated by octane oil, in glass capillaries of 150 *μ*m internal diameter^9^. For BZ droplet plugs of 1–7 nL volume and interdroplet octane plugs in the range of 100–400 *μ*m, stable anti-phase oscillation between neighbouring droplets or stationary Turing patterns were observed depending on the MA concentration, which was attributed to inhibitory droplet-droplet coupling facilitated by diffusion of bromine through the oil phase^9–11^. Coupling did not occur for interdroplet spacings >400 *μ*m because the diffusion time through the octane plugs then exceeds the period of the BZ oscillations^9–11^. Similar observations were made for nanoliter MA BZ droplets positioned in close proximity in circle and star droplet geometries^12,13^. Excitatory coupling was observed only at extremes of high and low malonic acid concentration, which determines the balance of oil-diffusable excitatory (BrO_2_, HBrO_2_) and inhibitory (Br_2_) reaction intermediates^14^.

In contrast, Thutupalli *et al*. brought surfactant-coated MA BZ droplets of ≈1 nL volume in direct contact, realising a 2D droplet geometry in 100 *μ*m to millimetres wide microfluidic channels with squalane as the oil phase^15,16^. Inhibitory coupling through oil gaps was not possible because the oil-solubilised surfactant also served as a bromine scavenger, but excitatory waves could propagate through the 2D BZ droplet network at droplet-droplet contact points, which was attributed to excitatory BZ species diffusing over an inter-droplet surfactant bilayer^15,16^. Guzowski *et al*. also observed coupling between microfluidically produced pairs of MA BZ droplets in hexadecane with asolectin lipids, manifested as larger droplets of ≈0.9 *μ*L volume increasing the inherent self-oscillation frequency of smaller droplets (down to ≈0.1 *μ*L), which are postulated to contain a lower concentration of oil-soluble reaction species because of a higher surface-to-volume ratio^17^. This coupling was explained by diffusion of excitatory species, specifically HBrO_2_, over the asolectin lipid bilayer at the droplet-droplet interface^17^. However, Torbensen *et al*. observed inhibitory coupling in a capillary-confined linear array of contacting MA BZ droplets, manifested as antiphase oscillations, with cyclohexane/chloroform containing synthetic phospholipids and an anionic detergent as the solvent phase^18^. Coupling over the lipid interface was almost completely abolished by the inclusion of cholesterol, postulated to react with the inhibitory bromine species, hence MA BZ droplets oscillated independently^18,19^.

We previously presented manually formed droplet networks with 1,4-cyclohexanedione (CHD) as the BZ substrate^20^. In this rapid prototyping approach, 3D-printed 1 mm wide trenches confined the position of pipetted ≈5 *μ*L CHD BZ droplets immersed in decane oil with asolectin lipids as surfactant. For linear and basic 2D arrays of up to 15 droplets, a reduced-to-oxidised transition of one droplet, manifested as a solid single wavefront traversing the droplet, could trigger a transition in the neighbouring droplet, hence propagating the CHD BZ excitation through the arrays^20^. There is a downside to the use of CHD BZ over MA BZ, however. The onset of visible oscillations takes up to several hours, rather than being instantaneous. The number of individual oscillation waves that can be observed before the medium is equilibrated is low and the excitation waves destabilise the droplet-droplet interface, leading to the merging of neighbouring droplets. There are three prerequisites for the formation of large functional droplet networks: stability of the droplets over an extended period, transmission of excitation over the inter-droplet interface, and a long lifetime of the excitability. BZ droplets-in-oil offer a wide scope for tuning droplet networks for the desired purpose. The interface can be adapted through the selection and combination of biological and synthetic surfactants^16,18^, while the reaction medium can be adapted by varying the concentration of its components over a range spanning orders of magnitude^21^. Accordingly the composition of BZ medium in published studies varies considerably. Nevertheless it has been a challenge to achieve stability for contacting BZ droplets-in-oil^22^, preventing systematic exploration of functional droplet networks.

We here focus on tuning the BZ medium with the aim of achieving large networks of coupled droplets which are in direct contact and possess distinct internal oscillation states. For a wide range of BZ compositions we determined wave characteristics over the complete lifetime of single MA BZ droplets of 2.5 mm diameter (≈5 *μ*L). However, it was subsequently found that these are not affected by contact with neighbouring droplets, regardless of their oscillation dynamics. Also, although the direction of the travelling wavefronts depends on the context provided by flanking droplets, the waves do not propagate across the droplet-droplet interface. We therefore explored the effect of adding CHD to the MA BZ medium. MA-CHD BZ droplets exhibited interdroplet wave propagation as previously observed for CHD BZ^20^, as well as stability and sustained oscillations typical for MA BZ. In combination these properties facilitate large networks of communicating BZ droplets which are stable over extended time periods, a requirement for the design of functional droplet architectures.

## Results and Discussion

### Single Droplets of Malonic Acid BZ

**Figure 1:**
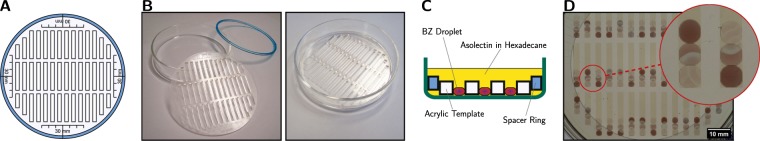
Recording of arrayed lipid-coated BZ droplets in oil. The droplets are localised with laser-cut acrylic templates (**A**) in a Petri dish (**B**) which is inclined 0.5° to let the droplets glide to the lower end of the 2.5 mm wide slots (**C**,**D**). This ensures both a defined droplet position for automated image processing and contact between droplets in experiments with multiple droplets per slot (**D** enlargement). All figures except Figs. 7 and 8B are orientated such that gravity pulls towards the lower edge. In the electronic version this and all subsequent figures can be enlarged for detail.

**Figure 2:**
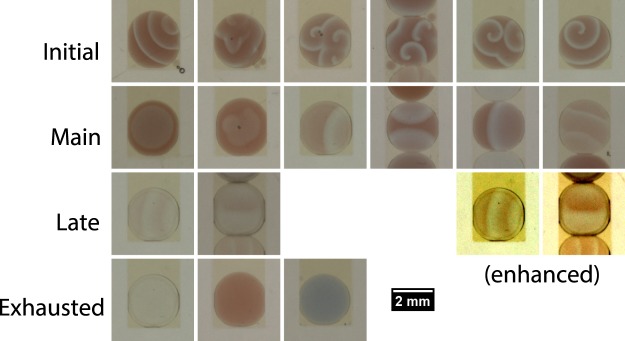
Samples of oscillation patterns that can occur over the lifetime of malonic acid BZ droplets. Immediately upon mixing the MA BZ reaction mixture enters the oscillatory period, which passes through the following distinct stages. *Initial period:* Low amplitude and high frequency planar or spiral waves, origins of waves can be identified. *Main period:* Rapid decrease in frequency from initial period, then frequency decreases only slowly over time. Typically there is only one origin of waves. The two leftmost panels show circular waves, the next four panels show planar waves. The rightmost panel shows a droplet with the relatively high frequency and low contrast that is also observed in droplets with low ferroin content. *Late period:* Characteristic is a sudden increase in frequency which then slowly decreases. The two rightmost panels are contrast-enhanced versions of the leftmost panels. Note that in the droplet with flanking droplets the wave travels upwards (from oxidised medium to reduced medium). *Exhausted state:* No oscillations detectable; droplets are typically transparent, but after the final oscillation the BZ medium can be in either the reduced (red) or oxidised (blue) state.

To explore a wide range of BZ compositions we use laser-cut templates to position droplets for parallel recording as illustrated in Fig. 1. The fixed position supports an automated image processing workflow to obtain the time evolution of individual droplets. The open oil-filled slots enable manual positioning of BZ droplets of any desired composition. We used the large number of slot positions to efficiently scope the BZ medium variants, including about five repeats for each condition. For single-droplet characterisation each slot holds one droplet and the Petri dish is imaged by a high resolution camera over several hours. Over this time the droplets develop through five distinct periods, four of which are shown in Fig. 2.

After preparing the reaction mixture a quiescent induction period of varying length^23^ precedes the onset of fast oscillations that mark the start of the initial period. The majority of the droplets begin oscillating with planar waves, however about 20% of the droplets start with spiral waves. The reaction then transitions into a long period of large-amplitude oscillations. This main period is the one relevant for wave propagation. Next is an abrupt increase in frequency that indicates the start of the late period. This is followed by the non-oscillatory exhausted state where the BZ medium is spent.

**Figure 3:**
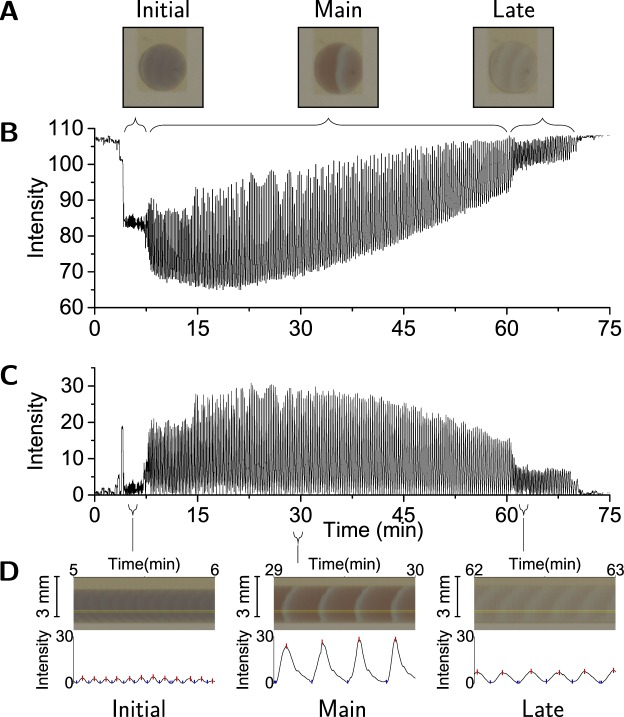
Extraction of wave features from video recording. (**A**) The depicted droplets are confined in the 2.5 mm wide slot (Fig. 1). Droplets are 108 pixels wide in the images and were recorded at 0.4 frames per second. For each video frame a 15 × 5 pixels region within the droplet is extracted, and its intensity determined (see [sec:org5a7951e]Methods). (**B**) The upper graph shows the temporal development of this intensity. (**C**) This intensity trace is then corrected for the baseline, yielding the lower graph. If all the 15-pixel wide slices from each video frame are composed horizontally a space time plot results. (**D**) One minute long sections of this plot are shown for the initial, the main and the late period; the 5-pixel high zone used to determine intensity is indicated in yellow. Their corresponding baseline-corrected intensities are shown below with the algorithmically identified peaks (red) and troughs (blue); these are then manually curated for assignment errors (see [sec:org5a7951e]Methods). The data shown in Fig. 4 is derived from the peaks and troughs such identified.

The video recording of the entire dish captures the droplets from pipetting a freshly prepared MA BZ solution into the oil-filled slots until exhaustion is observed. Here we consider the video cropped to a single droplet. For each video frame the blue-channel colour intensity of a region in each droplet is obtained, which yields the time development of the intensity amplitude. An example is shown in Fig. 3, indicating the initial, main, and late period. The different periods can be discerned from the time evolution of the intensity oscillations; for each period a sample frame of the droplet is shown. At the start of the record the droplet was not yet positioned and the clear oil registers as high intensity. After 4.5 minutes the droplet is pipetted into the slot and the fast, low amplitude oscillations of the initial period are visible in the graph (Fig. 3B). At 7.5 minutes the main period starts and lasts until 60 minutes, followed by a late period of about 10 minutes, after which the droplet is exhausted. Figure 3C shows the baseline-corrected amplitude used to quantify the wave features. Below the main graph, a panel shows for each period a one-minute section of the space-time plot obtained by laterally arraying a thin slice of each video frame. From this space-time plot the intensity development over time can be obtained, as shown in the lower half of the panel. The algorithmically determined peaks and troughs for the oscillation intensity, where each peak corresponds to a wavefront, are marked. Droplets of different compositions will give rise to a different time evolution of the redox state (cf. Supplementary Fig. S1).

**Figure 4:**
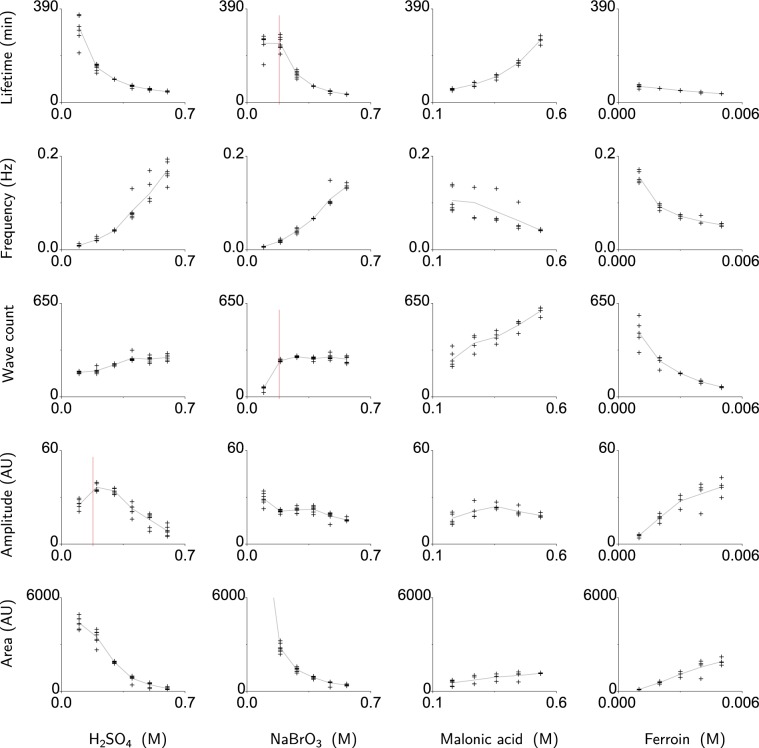
Characterisation of single malonic acid BZ droplets. The base composition of the BZ medium was: 0.5 M H_2_SO_4_, 0.47 M NaBrO_3_, 0.18 M malonic acid and 2 mM ferroin. In each column the concentration of one of these compounds was varied as indicated by the column label. For each concentration variant 4 to 7 repeats were performed, all are plotted on the graphs. The Lifetime was defined from the completion of mixing the medium to the start of the exhausted state. The Frequency is the median frequency of all individual oscillations over the lifetime. The Wave count is the peak count over the lifetime. Amplitude is the median of the intensity difference of the peaks and the baseline. The Area (arbitrary units, AU) is the median of the trough-to-trough integration of each peak (cf. Fig. 3) on the baseline-corrected intensity trace. At 0.09 M NaBrO_3_ the area exceeds double the area of the next higher concentration; not shown, mean: 10817 AU. Note the lowest concentration for both H_2_SO_4_ (0.1 M) and NaBrO_3_ (0.09 M) is below the malonic acid concentration (0.18 M), so the consumption of the latter is not dominating. Panels in which a vertical red line indicates the malonic acid concentration show a different trend for the H_2_SO_4_ and NaBrO_3_ concentration below and above 0.18 M.

We start from a base composition of 0.5 M H_2_SO_4_, 0.47 M NaBrO_3_, 0.18 M malonic acid and 2 mM ferroin and have explored variations in those concentrations that encompass a wide range of reaction mixtures found in the literature^24^ and which offer tunability of the droplet lifetime (Supplementary Figs. S2 and S3). To observe the effect of changing the concentration of each BZ chemical component, the process of observing and analysing the waves is repeated for each different BZ droplet composition. The concentration of one component of BZ is changed while other components are kept constant and the results are summarised in Fig. 4. According to previous studies, increasing the H_2_SO_4_ or NaBrO_3_ concentration should increase the wave frequency^25^ while increasing the MA concentration should increase the total number of oscillations and decrease the frequency at the end of the oscillation lifetime^26^. The data in Fig. 4 corroborate that increasing the H_2_SO_4_ and NaBrO_3_ concentration causes an increase in the oscillation frequency. The MA effect is also corroborated, where as MA concentration increases, the total number of oscillations increases and the mean frequency decreases (i.e. a longer wave period at the end of the oscillation lifetime). Increasing the ferroin concentration significantly reduces the lifetime, the oscillation frequency and the total number of oscillations.

### Malonic Acid BZ Droplets with Neighbours

The droplets used in the present study are large enough to exhibit distinct wave patterns within a single compartment. In conjunction with the wide concentration range under which the BZ reaction proceeds, this affords the study of interactions among droplets with identical or with different compositions. We focused on the same base composition as studied in the single-droplet experiments (0.5 M H_2_SO_4_, 0.47 M NaBrO_3_, 0.18 M malonic acid and 2 mM ferroin). Droplets of this composition can be brought into contact, however in over 150 arrays propagation into a neighbouring droplet was never observed.

Next, we investigated contacted droplets of different composition. If in adjacent droplets the H_2_SO_4_ or the NaBrO_3_ concentration is substantially lower than in the base composition, the interface between the droplets is unstable and the droplets have a high probability to fuse. Other variations of the base composition, giving different wave characteristics (Fig. 4), yield stable contacted droplets, but again interdroplet wave propagation is not apparent.

**Figure 5:**
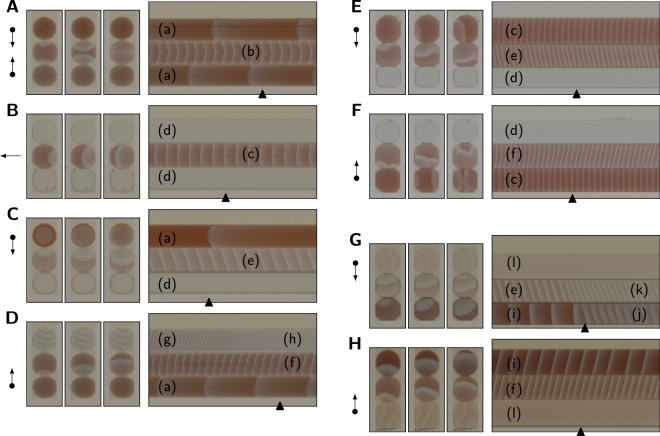
Neighbourhood effects in malonic acid BZ droplets with different compositions, corresponding to different oscillation lifetimes. In three-droplet structures the central droplet (‘sensor’), exposed to different combinations of relative lifetime of the two neighbouring droplets, is monitored. The left side of the sensor context panels depicts three snapshots of the droplet structure taken 5 s apart; an arrow indicates wave direction in the sensor, a dot at its origin marks the position of a longer lifetime droplet. On the right side are space-time plots (x = 10 min; y = 10 mm), the triangle indicates the time of the snapshots. The BZ composition is 0.5 M H_2_SO_4_, 0.47 M NaBrO_3_, 0.18 M malonic acid and 2 mM ferroin, with one component varied. (**A**–**D**) All four possible contexts with the NaBrO_3_ concentration being varied. Long-lifetime droplets (a) have 0.09 M, sensor droplets (b,c,e and f) have 0.28 M and short-lifetime droplets (d,g and h) have 0.47 M NaBrO_3_. Plots are taken at 60 min (**A**,**B**), 90 min (**C**), or 44 min (**D**) after mixing the medium. The wave pattern in the sensor droplet depends on the context. (**E**,**F**) Two contexts where the MA concentration is varied. Long-lifetime droplets (c) have 0.54 M, sensor droplets (e,f) have 0.36 M and short-lifetime droplets (d) have 0.18 M MA. Plots are taken at 60 min. Sensor wave direction depends on context. (**G**,**H**) Two contexts where the ferroin concentration is varied and the MA concentration is fixed at 0.36 M (i.e. doubled with respect to (**A**–**F**)). Short-lifetime droplets (i and j) have 5 mM, sensor droplets (e,f, and k) have 3 mM and long-lifetime droplets (l) have 1 mM MA. Plot (**G**) is taken at 83 min and (**H**) at 60 min. Sensor wave direction depends on context. (**A**–**H**) Main period wave patterns: (a) circular waves, (b) planar waves from both droplet interfaces meeting in middle of sensor droplet, (c,l) planar waves from the acrylic wall, (d) exhausted-state droplets, (e) planar waves from top to bottom interface, (f) planar waves from bottom to top interface, (g) and (i) planar waves. Late period wave patterns: high-frequency planar waves from droplet interface (h,j, and k).

Nevertheless, droplets with different composition in juxtaposition can influence their respective wave patterns as shown in Fig. 5 for three-droplet structures. We monitor the central (‘sensor’) droplet which is flanked by droplets that differ in composition from it. The wave pattern in the central droplet depends on the context provided by the flanking droplets. Initially, when both neighbours are oscillating in their main period, waves in the sensor tend to originate from the (opposing) sensor-neighbour droplet interfaces, with wavefront collision leading to wave annihilation (e.g. Fig. 5A). However, if the composition of the droplets differs in such a way that one neighbour has a shorter main-period lifetime than the opposing neighbour, waves from the interface with the longer-lifetime droplet tend to become dominant when the shorter-lifetime droplet approaches its exhausted state (see Supplementary Videos S1–S3). This applies to NaBrO_3_ (Fig. 5C,D) and MA (Fig. 5E,F) as well as ferroin (Fig. 5G,H) concentration variations. The figure panels show representative images from at least 5 repeat experiments for each NaBrO_3_ configuration, and from 16–19 repeats for each MA and ferroin configuration. When both neighbouring droplets have reached their exhausted state, waves in the sensor droplet originate from a contact point with the wall of the acrylic slot rather than a droplet-droplet interface (e.g. Fig. 5B).

**Figure 6:**
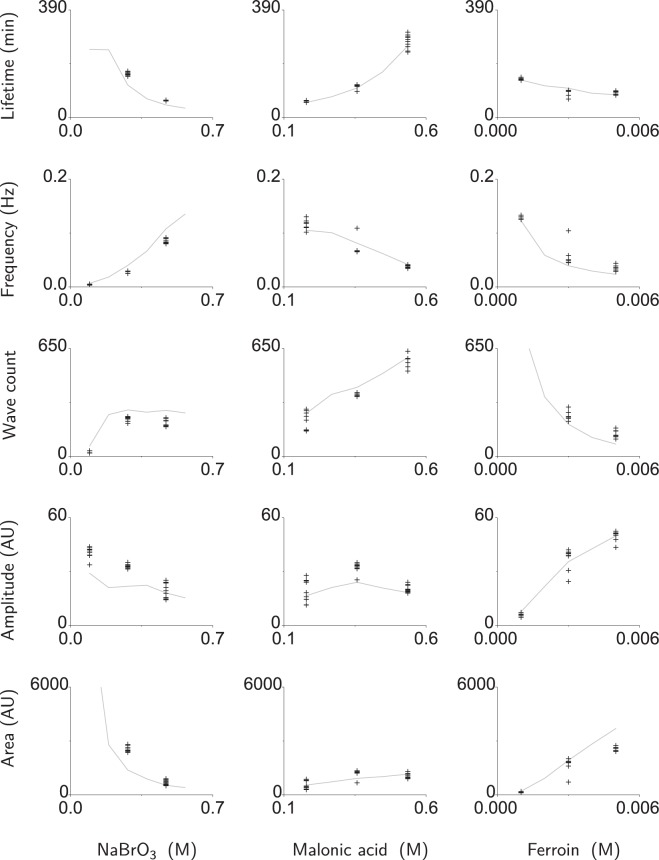
Characterisation of three-droplet arrays of malonic acid BZ with neighbours of different composition. Data markers correspond to arrayed BZ droplets, while grey lines connect data points for isolated droplets. All droplets have 0.5 M H_2_SO_4_, 0.47 M NaBrO_3_, 0.18 M MA and 2 mM ferroin unless otherwise stated. All droplets and array configurations correspond to those explored in Fig. 5. In the left column, NaBrO_3_ is varied to either 0.09, 0.28 or 0.47 M, with 12 values for each. This corresponds to three repeats where the centre 0.28 M droplet is flanked by droplets with 0.09 or 0.47 M NaBrO_3_ in all four possible configurations. The centre droplet can be flanked by two droplets of 0.09 M NaBrO_3_, or by two droplets of 0.47 M NaBrO_3_, or by 0.09 M NaBrO_3_ on the top and 0.47 M on the bottom side (or vice versa). The mean wave area for 0.09 M NaBrO_3_ is 22502 and 10817 (AU) for arrayed and isolated droplets, respectively. Note that for 0.09 M NaBrO_3_ the lifetime data is not available because the experiment was stopped when the sensor droplets were exhausted. The middle column shows characteristics of BZ droplets with only the MA concentration varied to either 0.18, 0.36 or 0.54 M, with 10 values for each. The 0.36 M centre droplet can be flanked by a droplet with 0.18 M MA on the top and 0.54 M on the bottom side, or vice versa, with five repeats for each configuration. The right column shows characteristics of BZ droplets where ferroin is varied to either 1, 3 or 5 mM, with 8 values for each. The MA concentration was doubled to 0.36 M to enable a sufficiently long lifetime of the oscillatory period for wave patterns in the centre droplet to stabilise. Droplets with 3 mM ferroin always occupied the centre position, and can be flanked by a droplet with 1 mM ferroin on the top and 5 mM on the bottom side, or vice versa, with four repeats for each configuration. The mean wave count for 1 mM ferroin is 936 and 803 for arrayed and isolated droplets, respectively.

It is also possible that the wave characteristics of any of the droplets in a three-droplet array are modulated by a neighbouring droplet when all droplets oscillate in their main period. To investigate whether such interdroplet coupling effects occurred, we quantified the key wave parameters for all droplet configurations shown in Fig. 5, using at least three repeats for each configuration. This analysis is presented in Fig. 6, where the data markers correspond to droplets in three-droplet arrays. It is clear that for each MA BZ composition, there is little spread in the obtained values, which is why data points in Fig. 6 were not individually labelled to indicate the configuration. For example, in the top left panel, a BZ droplet with the composition 0.47 M NaBrO_3_ could be positioned at the top or at the bottom of the array, while the droplet on the other side of the contacting 0.28 M NaBrO_3_ droplet could contain either 0.09 or 0.47 M NaBrO_3_. Yet for the four possible configurations of the array (Fig. 5A–D) the lifetimes obtained for different 0.47 M NaBrO_3_ droplets are very similar. Any more substantial variation in values, e.g. for the wave amplitude of the 0.47 M NaBrO_3_ mixture (Fig. 6), was similar to the variation observed in Fig. 4 for isolated droplets of identical BZ composition. Thus there is no strong correlation between the quantified wave characteristics and the position of a droplet in a three-droplet array, i.e. with a neighbour droplet of a lower or a higher NaBrO_3_, MA or ferroin concentration. The mean wave characteristics for isolated droplets (see Fig. 4 and Supplementary Fig. S3) are included in Fig. 6 as grey lines. It can be seen that for the investigated BZ compositions, the wave characteristics of a BZ droplet are similar when the droplet is isolated and when it is in contact with neighbours. Hence interdroplet coupling of oscillating MA BZ droplets of a range of compositions is not apparent. As previously noted, interdroplet wave propagation was also not observed.

### Coupled Droplet Networks

**Figure 7:**
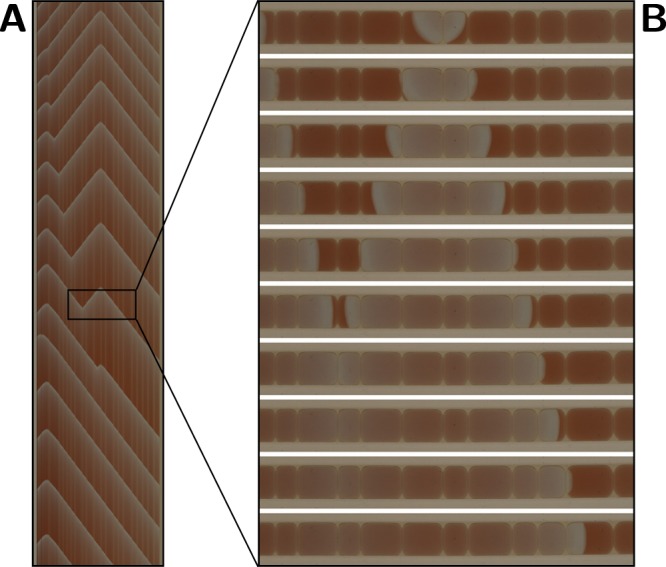
Propagation of mixed-substrate MA-CHD BZ waves through a 20-droplet array. An acrylic slot of 65 × 2.5 mm submerged in asolectin-hexadecane solution was filled with 30 droplets of a BZ medium that contained both malonic acid and 1,4-cyclohexanedione (0.09 M MA, 0.09 M CHD, 0.5 M H_2_SO_4_, 0.28 M NaBrO_3_ and 2 mM ferroin). After some initial droplet fusion events 20 droplets remained stable for the 9 h recording time. Wave transmission between droplets starts ≈30 min after mixing of the MA-CHD BZ medium. Oscillations were observed for ≈4 h. (**A**) A space-time plot for the same droplet array (57.5 mm for x-axis and 30 min for y-axis). Note that only a 15-pixel cross-section from individual image frames (such as the frames shown in the right panel) contributes to the space-time plot. Initially a wave emerged near the middle of the array, propagating towards the first and the last droplet of the array. When another wave emerged near the start of the array, it was able to propagate (after annihilation of the original wave source at ≈20 min in (**A**)) over ≈20 MA-CHD BZ droplets to the other side of the array. (**B**) A time series of a 12-droplet segment with 5 *μ*L and 10 *μ*L (fused) MA-CHD BZ droplets was taken at 10 s intervals. The left side represents droplets at the bottom of the acrylic slot, i.e. gravity pulls to the left in this panel. Waves characterised by wide wavefronts and multi-droplet wavelengths originate at two positions in the array and propagate from droplet to droplet. Wavefront collision leads to wave annihilation.

In a previous study on droplet-in-oil arrays wave propagation did occur when CHD was used as the reaction substrate, although only for a few oscillations because of the limited stability of the droplets in combination with the low oscillation frequency typical for CHD BZ^20,27^. We therefore decided to investigate the effect of adding CHD as a second reaction substrate to MA BZ mixtures. Mixed-substrate BZ systems, first described by Heilweil *et al*.^28^, can exhibit a complex evolution of the oscillation characteristics. For MA-CHD BZ this has been ascribed to ‘internal coupling’ of the reaction mechanisms of both substrates and to induction periods of different duration^27,29–31^. To assess droplet stability, we first brought droplets of MA-CHD BZ, containing 0.09 M of each substrate, into contact in a linear array of 30 droplets. As shown in Fig. 7, some droplet fusion occurred.

MA-CHD BZ waves characterised by wide wavefronts were observed, similar to waves in droplets of 100% CHD BZ^20^, reflecting the relatively slow oxidation of CHD^27^. Significantly, these travelling waves propagated from droplet to droplet, clearly seen in the space-time plot of Fig. 7, with waves originating near the start of the array travelling over ≈20 droplets (some of which enlarged due to fusion events) to the end of the array. Next, mixed-substrate BZ droplets with different molar ratios of CHD and MA with a total substrate concentration of 0.18 M were investigated. In arrays of three identical MA-CHD BZ droplets and a fourth droplet of pure MA BZ, 20% CHD (i.e. 80% MA) was sufficient for wave propagation through the MA-CHD BZ droplets, while ≥40% CHD also consistently enabled wave propagation into the BZ droplet with only 0.18 M MA as substrate.

For single MA-CHD BZ droplets of increasing CHD content, the intensity evolution was obtained as described for MA BZ droplets (e.g. Fig. 3). After an induction period, an initial period of fluctuating wave frequency was followed by a main period of a gradually declining, but not fluctuating, lower frequency and then by a late period of higher frequency. Example traces are included in Supplementary Fig. S1. In three-droplet arrays, wave propagation occurred only during the main period, suggesting that this is the period where CHD reactions dominate the oscillation pattern. A MA-CHD BZ medium with less than 50% CHD will immediately oscillate upon mixing and no induction period is observed. The time from mixing to the start of the main period is ≈25 min for 10% CHD (0.16 M MA), with similar values for a CHD content up to 40%. A medium with more than 50% CHD (i.e. less than 50% MA) will have an induction period that increases from 2 h for 60% CHD up to 3 h for 90% CHD. The mean frequency of the main period, derived from four isolated droplets for each MA-CHD BZ composition, was 0.04 s^−1^ for 0–50% CHD and reduced to ≈0.02 s^−1^ for 80–90% CHD. The mean duration of the main period was ≈1 h for 0–50% CHD, reducing to ≈45 min for 90% CHD, hence approximately 150 BZ waves could be observed during the main period, with interdroplet wave propagation enabled by ≥20% CHD. We selected a mixed-substrate composition of 50/50% MA-CHD because at a higher molar ratio of CHD the wavecount in the main period is reduced.

**Figure 8:**
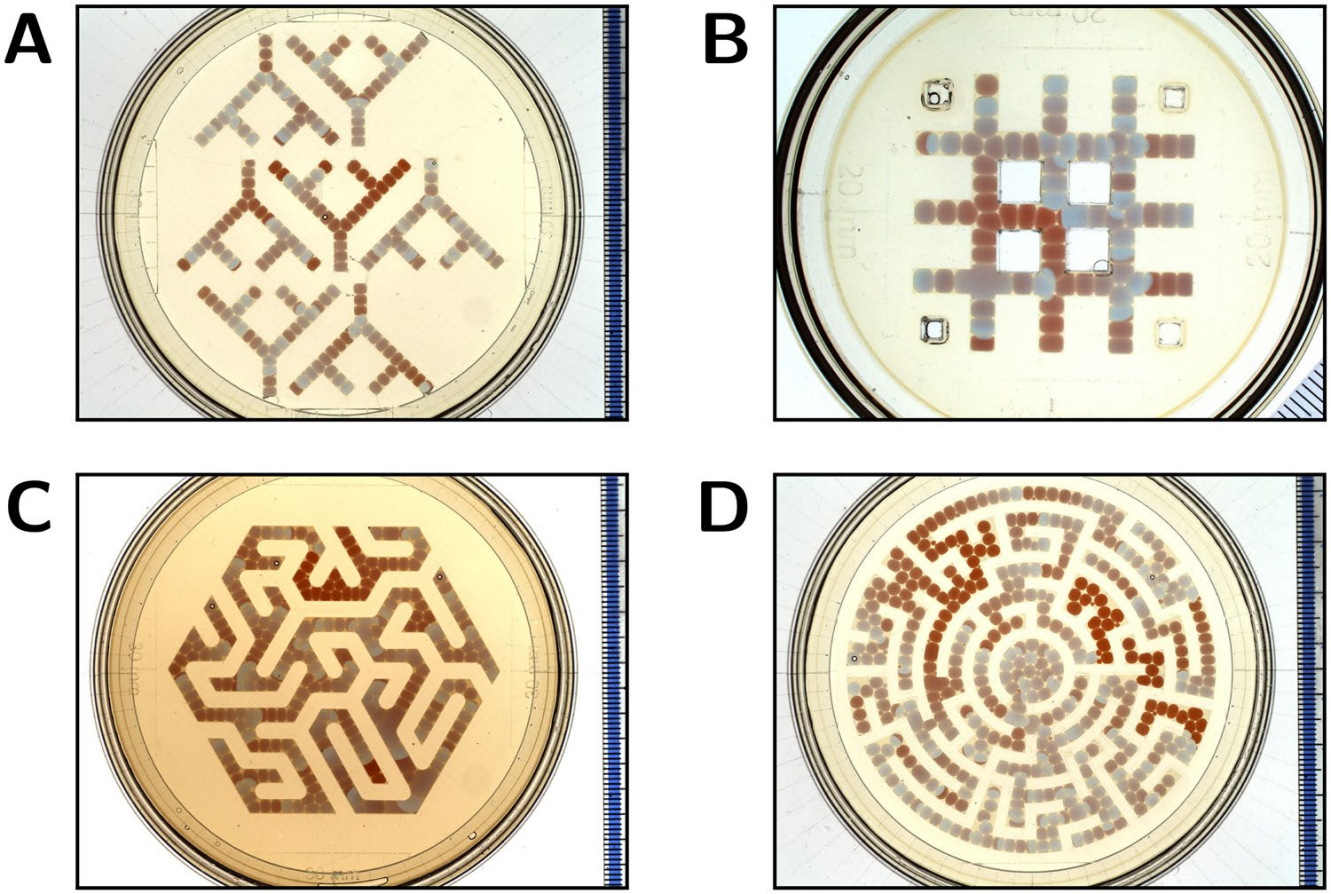
Networks of 50/50% MA-CHD BZ droplets of 5 *μ*L volume in an oil-lipid phase. (**A**) Branched linear arrays of ≈30 droplets. Droplets at the lower end of the arrays are more compressed due to the tilt angle of the Petri dish. At junctions waves can propagate in all directions and an incoming wave typically splits, exciting droplets in two downstream branches simultaneously. (**B**) Interconnected linear array of ≈80 droplets. During filling some droplets fused in the bottom left junction. At junctions waves can propagate in all directions and split over multiple paths. Over time an anticlockwise wave propagation was observed around the top-left enclosed square, with circulating waves triggering wave propagation in the other parts of the droplet network (Supplementary Video S4). (**C**) A hexagonal maze of linear segments containing ≈270 droplets, some of which fused during filling of the maze. At wider junctions that contain multiple droplets, waves propagate through all contacted downstream droplets as a single wavefront spanning multiple droplets. The oil phase appears darker than in the other panels because the hexadecane-asolectin solution was recycled from a previous experiment with 50/50% MA-CHD BZ droplets. (**D**) A circular maze of ≈400 droplets, 94% of which are stable after filling the maze and do not fuse for the duration of the experiment. In the central circle, a spiral wave spanning the ≈20 contacting droplets, emerged over time (Supplementary Video S5). In other parts of the maze, as for the other networks of self-oscillating MA-CHD BZ droplets (**A**–**C**), over time the wave sources become smaller in number and the wave frequency is reduced. The composition of the droplets is 0.5 M H_2_SO_4_, 0.28 M NaBrO_3_, 0.09 M MA, 0.09 M CHD and 4 mM ferroin. The diameter of the Petri dish in (**A**,**C**,**D**) is 90 mm, the dish in (**B**) is 60 mm.

To investigate wave propagation and droplet stability in large networks of coupled droplets, branched and interconnected network topologies of increasing size and with different junction geometries were laser cut in acrylic. The topologies used are: Y-shaped droplet arrays with three junctions able to accommodate ≈30 droplets (Fig. 8A), an interconnected linear array for ≈80 droplets (Fig. 8B), a hexagonal maze for ≈270 droplets (Fig. 8C) and a circular maze with a capacity of ≈400 droplets (Fig. 8D). The acrylic templates were immersed in oil with solubilised lipids and 5 *μ*L droplets of 50/50% MA-CHD BZ are pipetted into the channels. Droplet fusion may occur during filling, either because of insufficient time for lipids to assemble at the BZ-oil interface or due to droplet motion after formation of a droplet-droplet interface. The former is mitigated by pipetting the next droplet into a slot 1–3 mm away from the already present droplets and let it slide into position following the incline of the Petri dish. The latter is mitigated by using the spacer ring to fix the position of the acrylic template within the Petri dish. After any initial fusion events, droplet networks are stable for the duration of the oscillation period. The circular maze was left unperturbed overnight; this large droplet network was still stable 12 h after droplet filling.

All four networks (Fig. 8) exhibited wave propagation through all droplet interfaces during the main oscillation period, which lasts for at least 2 h, although some sections of the array oscillate up to 4 h. Waves can start at any position because the BZ medium is self-oscillatory. Typically, waves travel through the network, splitting at junctions into all paths downstream to the wave direction. Waves propagate until reaching a dead end or colliding with a wave arriving from the opposite side (i.e. until encountering droplets in the refractory state). Different parts of the larger networks can sustain oscillations of (somewhat) different frequency. Over time, the number of wave sources is reduced, enabling waves to travel over larger distances, and a few wave sources come to dominate the wave propagation pattern in the network (see Supplementary Videos S4 and S5).

It is observed that droplets contacted as a three-droplet triangle or as a multi-droplet cluster sometimes sustain a rotating wave which acts as a high-frequency wave source (Movie S4), suggesting that dominating wave sources can be established even when all droplets have the same self-oscillatory BZ composition. The wave propagation properties of the mixed-substrate BZ medium, in combination with the droplet stability of scalable networks, demonstrated here for nearly 400 droplet compartments, will enable systematic exploration of topology-directed wave pattern evolution in large droplet-in-oil networks.

## Conclusion

We introduce a new technique for studying oscillations in BZ droplets-in-oil that utilises laser-cut templates to localise either isolated or arrayed droplets at defined positions. This affords parallel recording of multiple experiments followed by automated image analysis. In contrast to the homogeneous droplet arrays described in the literature, this technique also enables the study of interactions among droplets of different BZ composition. We employed it here to efficiently probe the parameter space of BZ media for a composition suitable to implement droplet networks. This enabled us to identify the mixed-substrate MA-CHD BZ medium, the lifetime and propagation properties of which support the scale of networks that arguably will be required for demonstrating functional networks. The BZ medium has been extensively studied as an accessible model for complex chemical systems. In conjunction with the low cost and flexibility of network templates made by rapid prototyping it has the potential to also establish itself as a convenient research platform for functional droplet architectures. While this paper focuses on the characterisation of droplet behaviour as prerequisite to considering applications, our overall direction of research is motivated by the question whether artificial neuronal networks can potentially be implemented in soft matter rather than silicon. On a longer time-scale we envisage autonomous droplet architectures comprised of sensing, processing, and actuating droplets with shared chemical power supply and communication pathways. We anticipate that such a future droplet technology will exploit the flexibility of biochemical reaction networks and thus be biocompatible, opening new pathways not only in soft robotics but also in medicine.

## Methods

### Rapid prototyping

Poly(methyl methacrylate) (‘acrylic’) sheets of 2 mm thickness were patterned with an Epilog Mini 30 W CO_2_ laser cutter (Epilog Laser, CO, USA) to fit into a nominally 90 mm diameter Petri dish and to provide 2.5 mm wide slots for BZ droplet positioning, according to designs created in CorelDRAW Graphics Suite (Corel Corporation, Ottawa, Canada). Multiple linear slots were used for parallel characterisation of single BZ droplets and few-droplet arrays. Longer interconnecting slots were used for large droplet networks. The diameter of these acrylic templates was 4.2 mm smaller than the inner diameter of the glass Petri dish. An acrylic ring of 2 mm thickness was cut to fit along the curved inner dish edge, enabling the template to sit on the flat dish area (Fig. 1C). After laser cutting, acrylic templates and rings were flattened in a hot press at 1 tonne pressure (at least 15 min at 80 °C followed by 10 min at room temperature). The Petri dish was positioned in a laser cut holder, tilted at 0.5°, to facilitate alignment with the camera and droplet positioning at the lower edge of each slot. Before each experiment, the glass Petri dish was washed, sequentially, with deionised water, propanol and acetone, and dried with a nitrogen gas flow. Acrylic templates were wiped, sequentially, with tissue paper soaked in deionised water, propanol and again water, and then dried with dry tissue paper followed by a nitrogen gas flow.

### BZ preparation

Sulphuric acid H_2_SO_4_, malonic acid (MA) and 1,4-cyclohexanedione (CHD) were obtained from Sigma-Aldrich, 1,10-phenanthroline ferrous complex (ferroin) was from Fisher Scientific and sodium bromate NaBrO_3_ from Acros Organics. BZ mixtures were prepared in glass vials by sequentially pipetting stock solutions of H_2_SO_4_, NaBrO_3_, MA (or CHD) and ferroin (Supplementary Table S1). For the MA-CHD BZ mixture, CHD was added after the MA and the ferroin, because it was observed that the mixture usually failed to oscillate if CHD was added before the onset of MA BZ oscillations. The composition of MA BZ was 0.50 M H_2_SO_4_, 0.47 M NaBrO_3_, 0.18 M malonic acid and 2 mM ferroin, with variations as described in the Results section. Unless otherwise stated, the composition of the 50/50% MA-CHD BZ mixture was 0.50 M H_2_SO_4_, 0.28 M NaBrO_3_, 89 mM malonic acid, 93 mM CHD and 2 mM ferroin. With 4 mM ferroin the time to the start of wave propagation from droplet to droplet is up to 1 h shorter than with 2 mM.

### Droplet-in-oil formation

Asolectin lipid extract from soybean (≈25% phosphatidylcholine content) and hexadecane were obtained from Sigma-Aldrich. A solution of 38 mg/mL asolectin in hexadecane was prepared, allowing ample time for the lipids to dissolve on a rotary shaker. This solution was subsequently centrifugated for 15 min at 6,000 RCF and the small pellet of insoluble material was discarded. The Petri dish with the acrylic template was filled with 15 mL of the asolectin-hexadecane solution, taking care to avoid air bubbles, giving a ≈2 mm lipid-oil layer above the template’s surface. BZ droplets of 5 *μ*L volume were then pipetted into the oil-filled slots in the acrylic template, some distance away from either the lower edge of the slot or from an already present BZ droplet. The tilt angle of the dish ensured that the droplets moved slowly down the slot, allowing time for the lipids to assemble at the BZ-oil interface. For the few-droplet arrays, the time between droplet addition to the same slot was ≈2 min. Single-droplet BZ characterisation experiments were performed with a fresh asolectin-hexadecane solution. For droplet array experiments, the oil was typically recycled twice, by post-experiment separation of BZ and oil by centrifugation and storing the asolectin-hexadecane fraction at −20 °C. For the networks in Fig. 8A,B,D fresh oil was used for optimal stability.

### Imaging

The experiments, typically of 2–4 h duration, were recorded with a 3296 × 2472-pixel CCD camera (Prosilica GX3300C, Allied Vision Technologies, Germany) equipped with a 58 mm f/2 lens on a 12 mm extension tube, mounted above the Petri dish. The 2.5 mm slot width of the acrylic templates was defined by 108 pixels. Illumination was provided by a MiniSun A4 LED light pad below the dish. A curtain was placed over the camera and the observation area to minimise external light. VIMBA software (Allied Vision Technologies) was used to record BMP-format images of the entire dish at 0.4 frames/s and at a fixed exposure time (25 ms). These were converted to PNG format with ImageJ^32^ and assembled into an AVI video file.

### Quantitative analysis

For each of the 42 slots in a template, an individual space-time plot of a single droplet or of a three-droplet array was created by cropping along the centre of the slot a 15 pixel wide strip from every video frame, blurring it in the x-direction (across the 15 pixels), and placing these strips side by side in temporal order. Wave features were extracted from these space-time plots by selecting for each droplet in the slot a 5 pixel high strip across the time dimension (x-axis) at a y-position near the droplet centre and free of artifacts (e.g. CO_2_ bubbles, cf. Fig. 5, top droplet in panel A, and zones f–h in panel D.) throughout the observation time. This strip was then processed in Origin 9.1 (OriginLab Corporation, MA, USA) to low-pass filter, subtract the baseline comprised of the minimum intensity within a one period wide window, and finally obtain the amplitude information through manually adjusting Origin’s Peak Analyser and manually verifying that the peak assignments are correct. The frequency at a given time is determined as the inverse of the peak-to-peak time difference.

## Electronic supplementary material

Video S1

Video S2

Video S3

Video S4

Video S5

Supplementary Information

## Data Availability

All data supporting this study are openly available from the University of Southampton repository at 10.5258/SOTON/D0363.
