# Supplementary material for: Towards Functional Droplet Architectures: a Belousov-Zhabotinsky Medium for Networks
Source: Sci Rep. 2018 Aug 23;8:12656. doi: 10.1038/s41598-018-30819-6 (PMC6107623; doi:10.1038/s41598-018-30819-6)
Supplement: Supplementary file 6 — Supplementary Information [file 41598_2018_30819_MOESM6_ESM.pdf]

Supplementary Information

Towards Functional Droplet Architectures: a Belousov-Zhabotinsky Medium for Networks

Kai Ming Chang, Maurits R.R. de Planque and Klaus-Peter Zauner*

Electronics and Computer Science, University of Southampton, Southampton, SO17 1BJ, United Kingdom
*kpz@ecs.soton.ac.uk

TABLE S1. Belousov-Zhabotinsky (BZ) reaction mixture 2

FIGURE S1. Wave feature evolution depends on BZ composition 3

FIGURE S2. Effect of varying the MA concentration when the $NaBrO_3$ concentration is halved ... 4

FIGURE S3. Effect of varying the ferroin concentration when the MA concentration is doubled ... 5

VIDEO CAPTIONS S1–S5 6

Table S1. Belousov-Zhabotinsky (BZ) reaction mixture. Stock concentrations of the BZ reaction components are listed, with appropriate volumes to achieve specific concentrations in the complete reaction mixture. Deionised water is pipetted first into a 20 mL glass scintillation vial and then the various BZ components are added in the order indicated. A total reaction volume of 1 mL is obtained by starting with the appropriate volume of water. Note that 1,4-cyclohexanedione (CHD) is omitted for BZ solutions that only have malonic acid (MA) as substrate. For preparing mixed-substrate MA-CHD BZ, CHD is added 5 min after ferroin addition.

**Order**	**Component stock**		**Component in BZ reaction mixture**					
**1**	2.5 M H_2SO_4	Concentration (M)	0.1	0.2	0.3	0.4	0.5	0.6
		Added Volume (µL)	40	80	120	160	200	240
**2**	1.88 M $NaBrO_3$	Concentration (M)	0.09	0.19	0.28	0.38	0.47	0.56
		Added Volume (µL)	50	100	150	200	250	300
**3**	1.79 M malonic acid	Concentration (M)	0.09	0.18	0.27	0.36	0.45	0.54
		Added Volume (µL)	50	100	150	200	250	300
**4**	25 mM ferroin	Concentration (mM)	1	2	3	4	5	6
		Added Volume (µL)	40	80	120	160	200	240
**5**	0.93 M 1,4-cyclo-hexanedione	Concentration (M)	0.09	0.19	0.28	0.37	0.46	0.56
		Added Volume (µL)	100	200	300	400	500	600

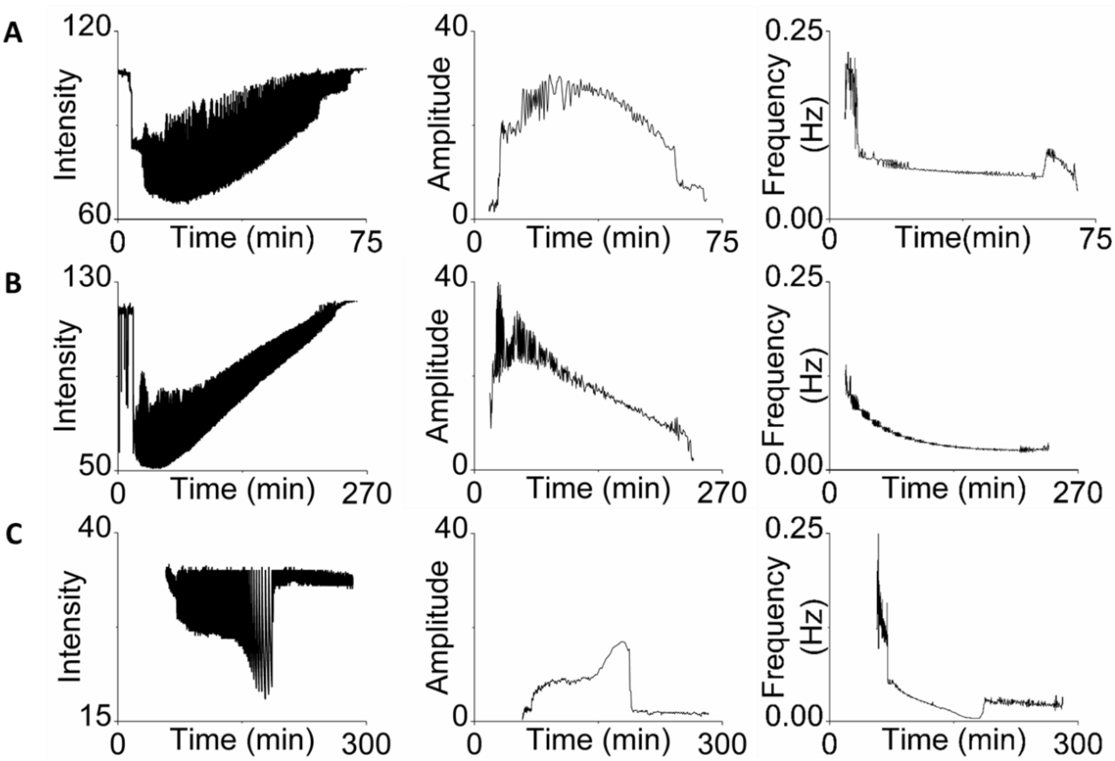

Figure S1. Wave feature evolution depends on BZ composition. For three different BZ droplet compositions, the pixel intensity measurement of the space-time plot, baseline-corrected amplitude and frequency evolution are shown. Time is measured from when the stock solution is mixed. Generally, when the droplet is pipetted into the oil, a short initial oscillation period with a high frequency and low amplitude of waves is observed. This is followed by a main period with low-frequency and high-amplitude waves, which lasts for a significant portion of the total oscillation lifetime. Amplitude and frequency are observed to decrease with time. The main period is followed by the late period, where the amplitude suddenly decreases and the frequency increases. (**A**) Medium-lifetime BZ droplet with 0.5 M H_2SO_4, 0.38 M $NaBrO_3$, 0.18 M MA, 2 mM ferroin corresponding to Fig. 3 in the main text. *Initial period:* 4.5 to 7.5 min, *main period:* 7.5 to 60.5 min, *late period:* 60.5 to 70 min. (**B**) Long-lifetime BZ droplet with 0.5 M H_2SO_4, 0.47 M $NaBrO_3$, 0.54 M MA, 2 mM ferroin. The initial period transitions gradually to the main period and then to the late period. Abrupt changes of wave frequency are not observed, but sudden changes in amplitude do occur. *Initial period:* 17 to 24 min, *main period:* 24 to 232 min, *late period:* 232 to 238 min. (**C**) 50/50% MA-CHD BZ droplet positioned in the middle of a three-droplet array where all droplets have the same composition (0.09 M MA, 0.09 M CHD, 0.5 M H_2SO_4, 0.28 M $NaBrO_3$ and 2 mM ferroin). Waves propagate through droplet-droplet interfaces during the main oscillation period, which is marked by an abrupt decrease in frequency. Unlike the pure-MA BZ droplets (A and B), during the main period the minimum pixel intensity becomes smaller, while the amplitude increases and the frequency is reduced. The wave count in the main period is also smaller compared with pure-MA BZ droplets due to a significantly lower oscillation frequency. *Initial period:* 58 to 71 min, *main period:* 71 to 187 min, *late period:* 187 to 283 min.

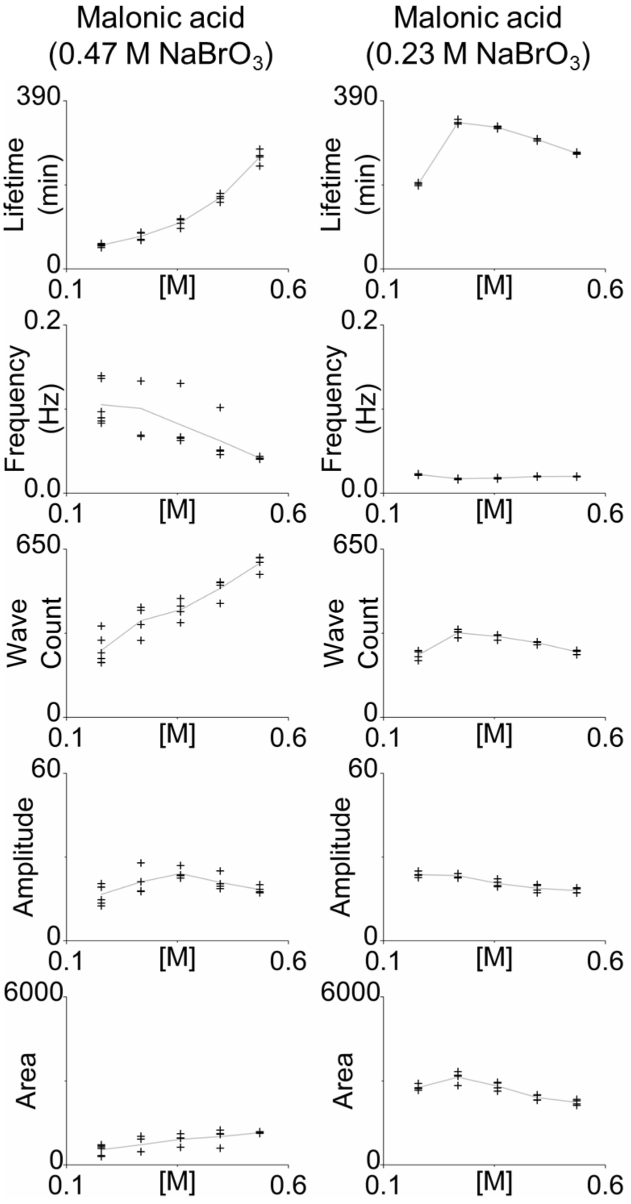

Figure S2. Effect of varying the MA concentration when the $NaBrO_3$ concentration is halved. This characterisation of single droplets is performed to explore whether droplets with a longer lifetime can be obtained by simultaneously increasing the MA concentration and decreasing the $NaBrO_3$ concentration. This is only successful when the MA concentration is < 0.27 M while the concentration of $NaBrO_3$ is 0.23 M. Due to this limited range of tuneable wave characteristics, 0.47 M $NaBrO_3$ is selected for the BZ base composition. For each concentration variant at least four repeat experiments were performed. The grey lines connect the mean values. All droplets contain 0.5 M H_2SO_4 and 2 mM ferroin; the left column is for 0.47 M and the right column for 0.23 M $NaBrO_3$. In both cases, the MA concentration is varied from 0.18 to 0.54 M. As the $NaBrO_3$ concentration is halved, the frequency reduces, the amplitude and the wave count remain similar, and the area increases. For 0.23 M $NaBrO_3$, raising the MA concentration above 0.27 M causes lifetime and wave count to decrease instead of increase. A similar trend is seen for droplets with 0.09 M $NaBrO_3$ (Fig. 4 in the main text; $NaBrO_3$ column): a reduced wave count with respect to higher $NaBrO_3$ concentrations and no further increase in oscillation lifetime despite the reduced $NaBrO_3$ concentration. Interestingly, for both cases, this deviation from the general trend occurs when the $NaBrO_3$ concentration is significantly lower than the MA concentration.

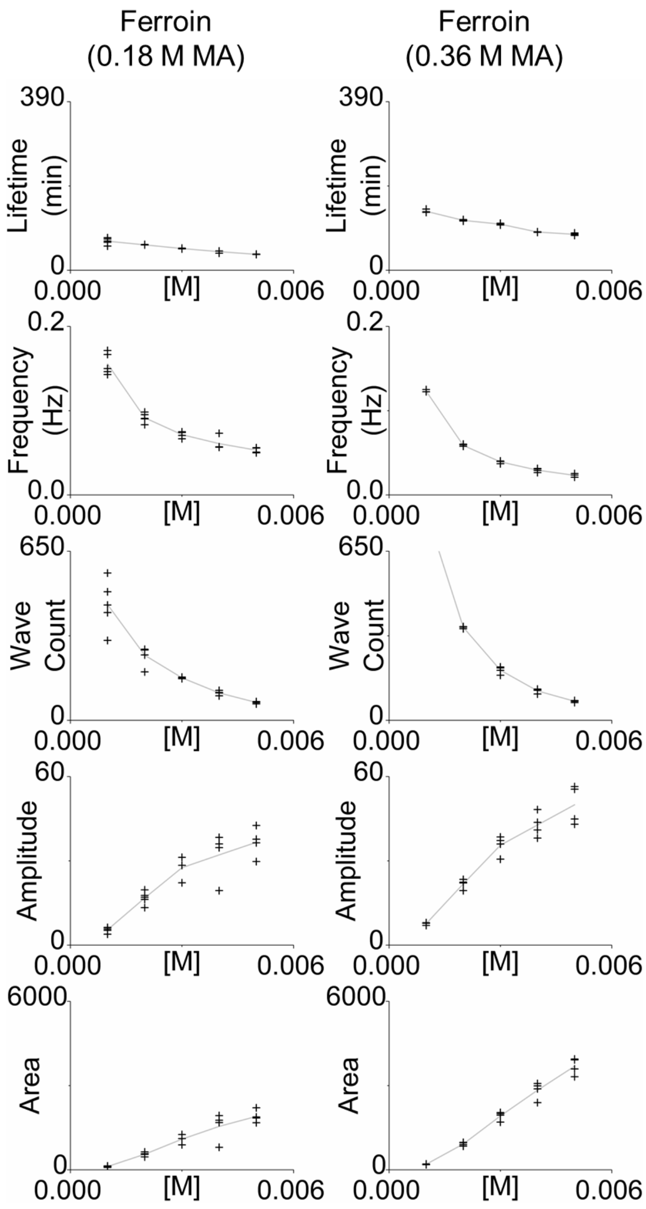

Figure S3. Effect of varying the ferroin concentration when the MA concentration is doubled. This characterisation of single droplets is performed to explore whether longer-lifetime droplets can be obtained by simultaneously reducing the ferroin and increasing the MA concentration. This is found to be tuneable for the investigated range of ferroin concentrations. For each concentration variant at least four repeats were performed. The grey lines connect the mean values. All droplets contain 0.5 M H_2SO_4 and 0.47 M $NaBrO_3$. The left column and right column correspond to droplets with 0.18 M and 0.36 M MA, respectively. The ferroin concentration is varied from 1 to 5 mM. When the MA concentration is doubled, the lifetime, wave count, amplitude and area are increased while the frequency is reduced. These trends are also seen in Fig. 4 in the main text (malonic acid column), consistent with the view that higher MA concentrations increase the wave count and lifetime with a relatively small effect on other wave characteristics, as compared with the effect of increasing the H_2SO_4, $NaBrO_3$ or ferroin concentrations (but note the different scenario for low $NaBrO_3$ concentration; Supplementary Fig. S2). This was exploited for the three-droplet arrays with different wave characteristics (Fig. 5 and Fig. 6 in the main text). The wave count for BZ droplets with 0.36 M MA and 1 mM ferroin is 803 waves.

Caption for Supplementary Video S1

Three-droplet arrays with $NaBrO_3$ concentration varied between droplets. All four configurations corresponding to Fig. 5A–D in the main text are shown. Interdroplet wave propagation is not apparent, but wave patterns in the middle 'sensor' droplets evolve according to the lifetime of the neighbouring droplets. The BZ composition, excluding $NaBrO_3$, is 0.5 M H_2SO_4, 0.18 M MA and 2 mM ferroin. From the left side to the right side of the video frame, the array topologies [bottom–centre–top] are: (D) = [(a)–(f)–(g, h)], (C) = [(d)–(e)–(a)], (B) = [(d)–(c)–(d)] and (A) = [(a)–(b)–(a)]. Here, (a) represents 0.09 M $NaBrO_3$, (b, c, e, f) 0.28 M $NaBrO_3$ and (d, g, h) 0.47 M $NaBrO_3$. Wavefronts in the centre (b, c, e, f) droplets all emerge from the interface with a longer-lifetime (a) droplet, starting at video time 0:46 min. An exception is the third array with no (a) droplets, where the waves start from the right-side acrylic wall. Wave characteristics for this experiment correspond to Fig. 6 ($NaBrO_3$ column graphs) in the main text. The video (frame size 20.4 × 9.0 mm) shows the BZ droplets from ≈34 to 82 min after BZ reaction mixture preparation and is accelerated such that 1 s in the video represents 30 s in real time. Each droplet is 5 µL in volume and 2.5 mm in diameter.

Caption for Supplementary Video S2

Three-droplet arrays with malonic acid (MA) concentration varied between droplets. Both configurations corresponding to Fig. 5E–F in the main text are shown. Interdroplet wave propagation is not apparent, but wave patterns in the middle 'sensor' droplets evolve according to the lifetime of the neighbouring droplets. The BZ composition, excluding MA, is 0.5 M H_2SO_4, 0.47 M $NaBrO_3$ and 2 mM ferroin. From the left side to the right side of the video frame, the array topologies [bottom–centre–top] are: (F) = [(c)–(f)–(d)], (E) = [(d)–(e)–(c)] and (F) = [(c)–(f)–(d)]. Here, (c) represents 0.54 M MA, (e, f) 0.36 M MA and (d) 0.18 M MA. Wave fronts in the centre (e, f) droplets all start from the interface with a longer lifetime (c) droplet, starting at video time 1:24 min. Wave characteristics for this experiment correspond to Fig. 6 (malonic acid column graphs) in the main text. The video (frame size 16.9 × 11.7 mm) shows the BZ droplets from ≈30 to 66 min after BZ reaction mixture preparation and is accelerated such that 1 s in the video represents 30 s in real time. Each droplet is 5 µL in volume and 2.5 mm in diameter.

Caption for Supplementary Video S3

Three-droplet arrays with ferroin concentration varied between droplets. Both configurations corresponding to Fig. 5G–H in the main text are shown. Interdroplet wave propagation is not apparent, but wave patterns in the middle 'sensor' droplets evolve according to the lifetime of the neighbouring droplets. The MA concentration is double that of Supplementary Video S1 and S2, so that droplets have a sufficiently long lifetime for wave pattern changes to be observed. The data for these compositions as isolated droplets is shown in Supplementary Fig. S3. The BZ composition, excluding ferroin, is 0.5 M H_2SO_4, 0.47 M $NaBrO_3$ and 0.36 M MA. From the left side to the right side of the video frame, the array topologies [bottom–centre–top] are: (H) = [(l)–(f)–(i)], (G) = [(i, j)–(e, k)–(l)] and (H) = [(l)–(f)–(i)]. Here, (l) represents 1 mM ferroin, (e, f, k) 3 mM ferroin and (i, j) 5 mM ferroin. Wavefronts in the centre (e, f, k) droplets all emerge from the interface with a longer lifetime (l) droplet, starting at video time 1:24 min. Wave characteristics for this experiment correspond to Fig. 6 (ferroin column graphs) in the main text. The video (frame size 17.0 × 12.2 mm) shows the BZ droplets from ≈31 to 61 min after BZ reaction mixture preparation and is accelerated such that 1 s in the video represents 30 s in real time. Each droplet is 5 µL in volume and 2.5 mm in diameter.

Caption for Supplementary Video S4

Interconnected linear arrays corresponding to Fig. 8B in the main text. This experiment is performed to investigate droplet networks with junctions and looped pathways, and to test if the refractory period prevents waves from propagating into sections upstream of the wave direction. All droplets are 50/50% MA-CHD droplets, which have 0.09 M MA, 0.09 M CHD, 0.5 M H_2SO_4, 0.28 M $NaBrO_3$ and 4 mM ferroin. To produce droplet networks with looped pathways around square pillars, free-standing pillar structures, unconnected to other parts of the acrylic template are needed. Therefore, a foundation acrylic template layer is fabricated that contains holes for pillar positioning. Note that this implies that the droplets rest on acrylic instead of glass, as in all other experiments. During filling, some droplets fused in the bottom-left junction. Typically, waves travel through the network, splitting at junctions into all paths downstream to the wave direction. Waves propagate until reaching a dead end or colliding with a wave arriving from the opposite side (i.e. until encountering droplets in the refractory state). Over time, the number of wave sources is reduced, enabling waves to travel over larger distances, and a few wave sources come to dominate the wave propagation pattern in the network. A wavefront that enters multiple droplets propagates as a single wavefront spanning all those droplets. Some interfaces, such as in the top-middle of the network, temporarily cannot propagate waves. The top-middle junction initially acts as a dominating wave source, where waves with a higher frequency are formed, resembling a spiral wave encompassing multiple droplets. After some time, an anticlockwise wave propagation was observed around the top-left enclosed square, with circulating waves triggering wave propagation in the other parts of the droplet network. BZ droplets oscillate for ≈3 h, although some sections oscillate up to ≈4 h. The network is stable while left overnight; droplet fusions only occur during the initial droplet positioning (array filling) procedure. The video (frame size 49.1 × 35.7 mm) shows the BZ droplets from ≈124 to 136 min after BZ reaction mixture preparation and is accelerated such that 1 s in the video represents 30 s in real time. Each unfused droplet is 5 µL in volume and ≈2.5 mm in diameter.

Caption for Supplementary Video S5

Circular maze network corresponding to Fig. 8D in the main text. This experiment is performed to investigate a larger network of droplets with more junctions but without looped pathways. All droplets are 50/50% MA-CHD droplets, which have 0.09 M MA, 0.09 M CHD, 0.5 M H_2SO_4, 0.28 M $NaBrO_3$ and 4 mM ferroin. The circular maze is a single large network without loops but with many junctions and a centre section consisting of many contacted droplets. Gravity acts towards the bottom of the video frame. Typically, waves travel through the network, splitting at junctions into all paths downstream to the wave direction. Waves propagate until reaching a dead end or colliding with a wave arriving from the opposite side (i.e. until encountering droplets in the refractory state). Over time, the number of wave sources is reduced, enabling waves to travel over larger distances, and a few wave sources come to dominate the wave propagation pattern in the network. A wavefront that enters multiple droplets propagates as a single wavefront spanning all those droplets. In the central circle of the maze there is a higher wave frequency. Here, droplets excite each other as an interconnected multi-droplet cluster, resembling a spiral wave spanning the ≈20 contacting droplets. This is also observed in interconnected droplet clusters at maze junctions. BZ droplets oscillate for ≈2 h, although some sections of the array oscillate up to ≈4 h. The network is stable while left overnight; droplet fusions only occur during the initial droplet positioning (array filling) procedure. The video (frame size 76.8 × 76.2 mm) shows the BZ droplets from ≈137 to 143 min after BZ reaction mixture preparation and is accelerated such that 1 s in the video represents 30 s in real time. Each unfused droplet is 5 µL in volume and ≈2.5 mm in diameter.
